# Integrated Analysis of the Transcriptome and Metabolome Revealed Candidate Genes Involved in GA_3_-Induced Dormancy Release in *Leymus chinensis* Seeds

**DOI:** 10.3390/ijms22084161

**Published:** 2021-04-17

**Authors:** Bing Li, Pan Zhang, Fengdan Wang, Ran Li, Jian Liu, Qiannan Wang, Wei Liu, Bo Wang, Guofu Hu

**Affiliations:** 1 College of Animal Science and Technology, Northeast Agricultural University, Harbin 150030, China; libing253661949@163.com (B.L.); zhangpan@neau.edu.cn (P.Z.); wangfengdan122@163.com (F.W.); liran088@163.com (R.L.); wangqiannan@singleronbio.com (Q.W.); liuwei961117@163.com (W.L.); neauwangbo1996@163.com (B.W.); 2 Institute of Grassland and Ecology, Jilin Academy of Agricultural Sciences, Changchun 130033, China; liujian556677@163.com

**Keywords:** *Leymus chinensis*, seed dormancy, transcriptome, metabolome, GA_3_, dormancy release

## Abstract

*Leymus chinensis* is a perennial forage grass that has good palatability, high yield and high feed value, but seed dormancy is a major problem limiting the widespread cultivation of *L. chinensis*. Here, we performed transcriptomic and metabolomic analysis of hulled and de-hulled seeds of *L. chinensis* treated with or without GA_3_ to investigate the changes in gene and metabolites associated with dormancy release induced by GA_3_. The germination test revealed that the optimum concentration of GA_3_ for disruption of *L. chinensis* seed dormancy was 577 μM. A total of 4327 and 11,919 differentially expressed genes (DEGs) and 871 and 650 differentially abundant metabolites were identified in de-hulled and hulled seeds treated with GA_3_, respectively, compared with seeds soaked in sterile water. Most of the DEGs were associated with starch and sucrose metabolism, protein processing in the endoplasmic reticulum, endocytosis and ribosomes. Furthermore, isoquinoline alkaloid biosynthesis, tyrosine metabolism, starch and sucrose metabolism, arginine and proline metabolism, and amino sugar and nucleotide sugar metabolism were significantly enriched pathways. Integrative analysis of the transcriptomic and metabolomic data revealed that starch and sucrose metabolism is one of the most important pathways that may play a key role in providing carbon skeletons and energy supply for the transition of *L. chinensis* seeds from a dormant state to germination by suppressing the expression of *Cel61a*, *egID*, *cel1*, *tpsA*, *SPAC2E11.16c* and *TPP2*, enhancing the expression of *AMY1.1*, *AMY1.2*, *AMY1.6* and *GLIP5*, and inhibiting the synthesis of cellobiose, cellodextrin, and trehalose while promoting the hydrolysis of sucrose, starch, cellobiose, cellodextrin, and trehalose to glucose. This study identified several key genes and provided new insights into the molecular mechanism of seed dormancy release induced by GA_3_ in *L. chinensis*. These putative genes will be valuable resources for improving the seed germination rate in future breeding studies.

## 1. Introduction

Seed dormancy is defined as the failure of an intact viable seed to complete germination under optimal conditions [1]. It is a property formed in the last stage of seed development and is an adaptation mechanism that prevents seed germination in unfavourable environments or regulates when and where the seeds germinate [2,3,4]. This dormant state helps to maintain seed vigour until germination. Although seed dormancy can prevent germination, which is advantageous for storage after harvest, this state is unfavourable for crop cultivation. Understanding the mechanisms of dormancy and the methods of dormancy release, as well as regulating the transition of crop seeds from dormancy to germination, is extremely important for agricultural production [5].

Seed dormancy is mainly divided into five types: physiological dormancy, morphological dormancy, morphophysiological dormancy, physical dormancy and combinational dormancy (physiological + physical dormancy) [2]. It is generally believed that physiological dormancy is the most common type in grasses (Poaceae) and is caused by impairments in the metabolism of seeds, which may be related to hormone content or balance [6]. Dormancy and germination are two complex biological processes that are influenced by many genetic and environmental factors, such as endogenous hormones, light, moisture and temperature. For seeds of some species, the optimal germination temperature ranges from 15 to 20 °C, and higher temperatures lead to inhibition of germination [7]. It was reported that freshly harvested seeds of *Arabidopsis thaliana* (ecotype Col-0) are dormant and do not germinate in darkness at 25 °C, but they germinate well under constant white or blue light conditions, or when stored for 5 weeks at 20 °C, which also indicates that irradiation with white or blue light can alleviate the inhibitory effect of temperature on seed germination [8,9]. However, this promoting effect of blue light on dormancy release in dormant seeds can be significantly reduced by abscisic acid (ABA) treatment [9]. The essential component of light-induced germination is phytochrome-mediated signalling pathways, which can convert received light signals to internal cues and regulate genes related to gibberellin (GA)/ABA metabolism and signalling pathways, resulting in a decrease in ABA content and an increase in GA content [9,10]. PIF3–LIKE 5 (PIL5) is one of the interacting proteins of phytochromes that regulates GA and ABA metabolism, which plays a negative role in seed germination. Mutant *pil5* seeds can germinate well even in darkness accompanied by increased endogenous GA_4_ levels, whereas the germination of *pil5*-overexpressing seeds requires higher light irradiation [11].

In the context of hormonal balance, ABA and GA are a pair of very important hormone molecules that play antagonistic roles in the regulation of seed germination and dormancy [12]. ABA induces the establishment of seed dormancy during seed maturation and maintains this state, while GA promotes dormancy release and germination [13], and the interaction between ABA and GA during the metabolism and signal transduction process determines the final status of dormancy and germination in the seed [14]. Some related genes involved in the biosynthesis, antagonism and signalling pathways of GA and ABA have already been identified in many plants. DELLA proteins in the GA signalling pathway are negative regulators of dormancy release. *GIBBERELLIN INSENSITIVE DWARF 1* (*GID1*) can interact with DELLA proteins to form a GAGID1-DELLA complex in the presence of GAs, and this complex is then degraded, resulting in the activation of the GA signalling pathway [15,16]. Transcriptomic analysis found that transcript levels of the *GID1* family related to the GA pathway were upregulated during the dormancy release stages in grapes, while the levels of DELLA family members were downregulated [17]. In the process of GA-mediated promotion of seed germination, *GA3ox1* and *GA3ox2* are two key genes regulating GA synthesis [18], while *GA2ox* promotes GA degradation [19]. *REVEILLE1* (*RVE1*) is a Myb-like transcription factor involved in seed dormancy and germination; it can interact with *REPRESSOR OF GA-LIKE2* (*RGL2*) to regulate the dormancy and germination of *Arabidopsis thaliana* seeds by integrating light perception, GA metabolism and the associated signalling pathways [20] and repress GA biosynthesis by directly inhibiting the expression of *GA3ox2* [21]. In addition to these gene regulatory effects, metabolite levels are also correlated with seed germination. During the transition of seeds from a dormant state to germination, carbohydrate metabolism and plant hormone signal transduction pathways are activated [22]. It was reported that exogenous GA_4_ may play an important role in dormancy release by changing the abundances of metabolites involved in galactose, glyoxylate, dicarboxylate and starch and sucrose metabolism [23]. Thus, the change from a dormant state to germination in seeds is a complex process affected by both genes and metabolites.

*Leymus chinensis*, also known as alkali grass, is a perennial rhizome grass of *Leymus* Hochst. and is an important forage and soil and water conservation plant. This grass has important economic and ecological value due to properties such as cold tolerance, drought tolerance, salt tolerance and trampling tolerance. In the natural environment, *L. chinensis* is dominated by asexual propagation due to its long seed dormancy period and low germination rate, which has greatly restricted its extensive application in artificial grassland construction and degraded grassland restoration. Some studies have suggested that mechanically tied lemmas and seed coats prevent the infiltration of accelerators (GA, 6-benzyladenine, naphthalene acetic acid, etc.) and the exudation of inhibitors (citric acid, malic acid, ABA, etc.), leading to seed dormancy of *L. chinensis*; in addition, the large amount of ABA in the lemmas, seed coats and endosperm is also a factor inhibiting seed germination [24,25,26]. Furthermore, treating seeds with variable temperatures can promote germination to a certain extent, and the transcriptomic data of *L. chinensis* seed germination at variable temperatures showed that the genes related to seed germination were *Chi1*, *CBF3*, *GA3ox*, *EXPB4* and *SAIN1* [27]. With the development of animal husbandry and the strengthening of ecological environment management, there is an increased demand for improved seed quantity and quality of *L. chinensis* [28,29]. Therefore, dormancy release and improvement of the germination rate of *L. chinensis* seeds has become a hot topic, and the application of GA_3_ to enhance seed germination has provided new opportunities for the production of *L. chinensis*. Although studies on the physiological mechanism by which GA_3_ increases the germination rate of *L. chinensis* seeds have been reported, little attention has been given to the molecular mechanism at the gene level.

In this study, the optimal concentration of GA_3_ was first selected from hulled and de-hulled seeds of *L. chinensis*. Furthermore, an integrated transcriptomic and metabolomic analysis of hulled and de-hulled seeds of *L. chinensis* treated with or without GA_3_ was performed to identify important pathways, candidate genes and metabolites, and to identify the regulatory networks involved in GA_3_-induced dormancy release. Our findings provide new insight into how GA_3_ promotes seed dormancy release and will be helpful for improving the seed germination rate in *L. chinensis* breeding.

## 2. Results

### 2.1. Effect of GA_3_ on the Germination Rate, Germination Index and Germination Potential of Leymus Chinensis Seeds

**Figure 1:**
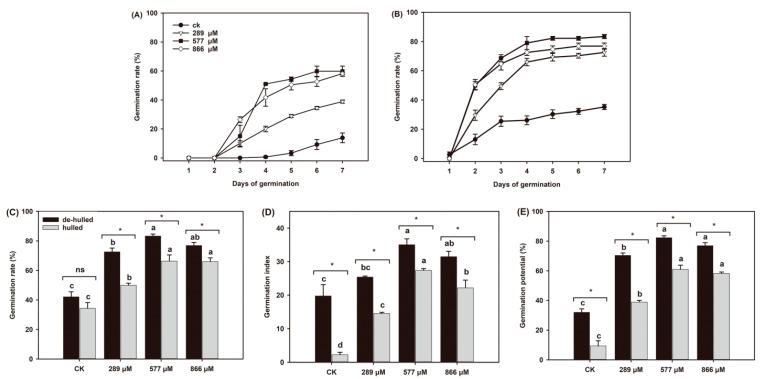
Effects of GA_3_ on the germination characteristics of *L. chinensis* seeds. (**A**) Germination rate of hulled seeds in the first 7 days. (**B**) Germination rate of de-hulled seeds in the first 7 days. (**C**) Total germination rate at 21 days. (**D**) Germination index. (**E**) Germination potential. Different letters indicate significant differences between different concentrations of GA_3_ according to Duncan’s multiple comparisons test (*p* < 0.05). Asterisks (*) indicate significant differences between de-hulled and hulled seeds according to the *t*-test (*p* < 0.05).

Compared to the control treatment, three concentrations of GA_3_ significantly promoted germination of *L. chinensis* seeds, either hulled or de-hulled, and the hulls that covered the seeds inhibited germination, leading to a delayed initial germination time and a decreased germination rate (Figure 1). The initial germination time of de-hulled seeds was day 2, while hulled seeds treated with or without GA_3_ began to germinate on day 3 and day 4 (Figure 1A,B). The total germination rate, germination index and germination potential of de-hulled seeds were higher than those of hulled seeds, and both reached their maximum values after treatment with 577 μM GA_3_, with the values increasing by 97.98%, 77.47% and 157.03%, respectively, compared to those of the control (Figure 1C–E).

### 2.2. Transcriptomic Analysis of L. chinensis Seeds Treated with GA_3_

**Figure 2:**
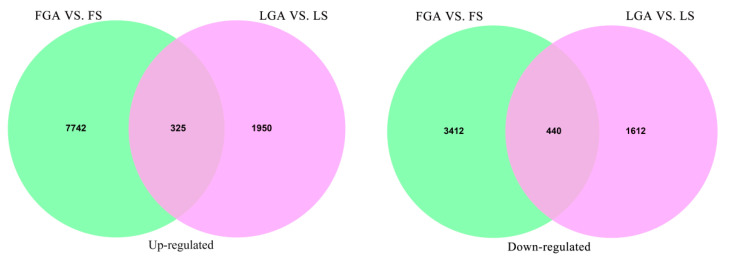
Up- and downregulated genes in *L. chinensis* seeds under different treatments. LGA: de-hulled seeds soaked in 577 μM GA_3_ solution for 24 h; LS: de-hulled seeds soaked in sterile water for 24 h; FGA: hulled seeds soaked in 577 μM GA_3_ solution for 24 h; FS: hulled seeds soaked in sterile water for 24 h.

We generated a total of 118.22 Gb of valid bases with Q30 values ranging from 95.27~96.72%, and the mean GC content was 54.06% (Table S1). After de novo assembly with the Trinity package, we obtained a total of 203,776 transcripts and 37,208 genes, with GC contents of 48.96% and 49.03%, respectively. The N50 of the genes was 1541, the total number of assembled bases was 40,055,874 (Table S2), and the maximum, minimum and median lengths of the genes were 19,928, 201 and 806, respectively. We adopted the criteria |log_2_FC| > 1 and false discovery rate (FDR) ≤ 0.05 to screen differentially expressed genes (DEGs) in hulled and de-hulled seeds treated with GA_3_ and distilled water. A total of 4327 DEGs of de-hulled seeds soaked in 577 μM GA_3_ solution for 24 h (LGA) vs. de-hulled seeds soaked in sterile water for 24 h (LS) were screened out, of which 2275 genes showed upregulated expression and 2052 genes showed downregulated expression (Figure 2 and Table S3). Moreover, 11,919 DEGs of hulled seeds soaked in 577 μM GA_3_ solution for 24 h (FGA) vs. hulled seeds soaked in sterile water for 24 h (FS) were screened from the hulled seeds, of which 8067 were upregulated and 3852 were downregulated. In addition, 325 upregulated genes and 440 downregulated genes among these genes were coexpressed in both LGA vs. LS and FGA vs. FS.

Gene Ontology (GO) analysis was performed in this study to analyse the functions of the DEGs (*p* < 0.05). From the data shown in Table S4, a total of 6913 and 2378 genes were annotated in three GO functions in FGA vs. FS and LGA vs. LS, respectively. In the biological process category, oxidation-reduction process (GO: 0055114, 341 genes), metabolic process (GO: 0008152, 292 genes) and translation (GO: 0006412, 290 genes) were the most enriched in the FGA vs. FS comparison, while the top three enriched GO terms in LGA vs. LS were biological process (GO: 0008150, 254 genes), oxidation-reduction process (GO: 0055114, 121 genes) and response to cadmium ion (GO: 0046686, 64 genes). There were 362 and 219 genes enriched in the cellular component category (GO: 0005575) in FGA vs. FS and LGA vs. LS, respectively. Furthermore, metal ion binding (GO: 0046872, 453 genes), nucleotide binding (GO: 0000166, 364 genes) and structural constituent of ribosome (GO: 0003735, 328 genes) were the top three enriched terms in FGA vs. FS, and molecular function (GO: 0003674, 287 genes); hydrolase activity, hydrolysing O-glycosyl compounds (GO: 0004553, 37 genes), and peroxidase activity (GO: 0004601, 35 genes) were the top three terms in LGA vs. LS.

**Figure 3:**
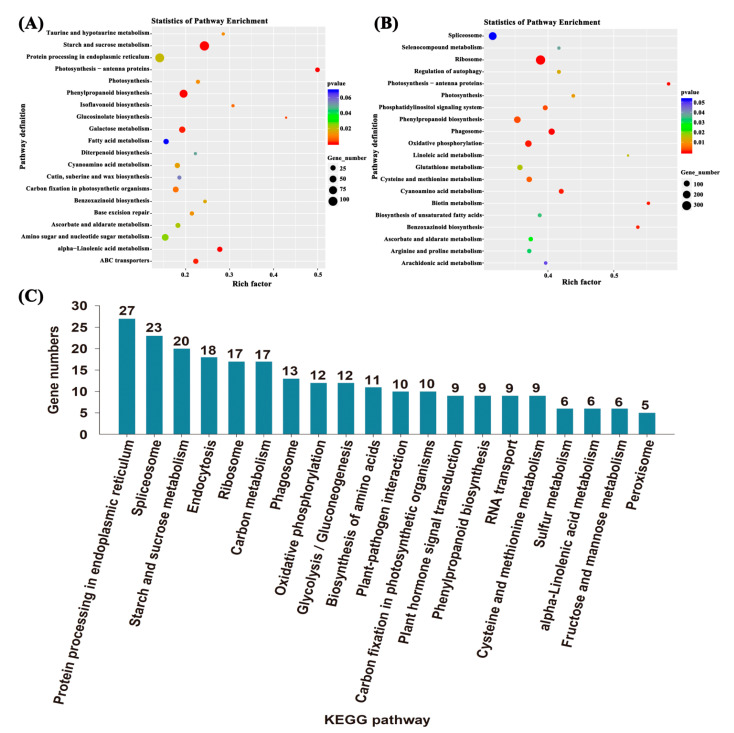
KEGG pathway enrichment analysis of DEGs in *L. chinensis* seeds under different treatments. (**A**) LGA vs. LS; (**B**) FGA vs. FS; (**C**) DEGs with similar regulatory trends significantly enriched in both LGA vs. LS and FGA vs. FS. The size of the circles represents the number of genes enriched in the pathway, and the colour of the circle represents the *p* value.

Kyoto Encyclopedia of Genes and Genomes (KEGG) pathway enrichment analysis revealed that the DEGs in LGA vs. LS seeds of *L. chinensis* were mainly related to starch and sucrose metabolism, phenylpropane biosynthesis, sugar metabolism, α-linolenic acid metabolism, ABC transporter and photosynthesis proteins (Figure 3A), while the DEGs in FGA vs. FS were enriched in ribosome, phenylpropane biosynthesis, phagocytosis, energy metabolism, amino acid metabolism and phosphatidylinositol signalling system (Figure 3B). The DEGs with similar regulatory trends in both LGA vs. LS and FGA vs. FS were also screened, and these genes were mainly enriched in protein processing in the endoplasmic reticulum, spliceosome, starch and sucrose metabolism, endocytosis and ribosome (Figure 3C).

### 2.3. Validation of RNA-Seq Data by qRT-PCR

**Figure 4:**
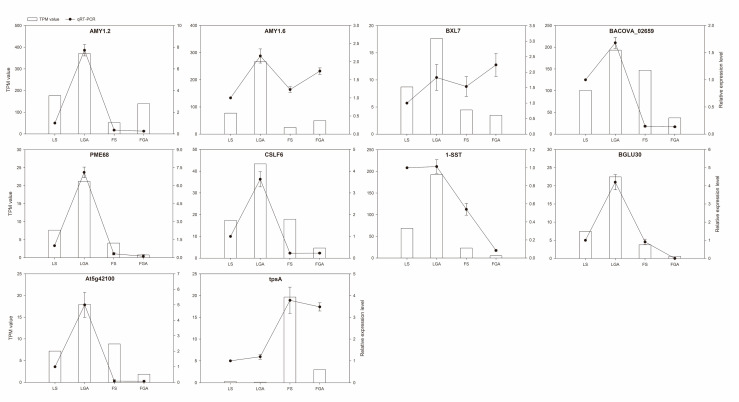
Validation of the relative expression levels of candidate unigenes by RT-qPCR. The left *y*-axis represents the TPM value, and the right *y*-axis represents the relative expression level. Lines represent the means ± SDs (*n* = 3).

To further determine the accuracy of the RNA sequencing results, ten DEGs involved in *L. chinensis* seed dormancy release were selected for qRT-PCR, and specific primers for these genes were designed by Primer 6.0 software (Table S5). The RNA sequencing results showed that the expression levels of 9 of the 10 DEGs were significantly upregulated in LGA compared to LS, and the expression levels of 7 of the 10 DEGs were significantly downregulated in FGA compared to FS. The qRT-PCR results were largely consistent with RNA seq data, which proved that the transcriptome sequencing data for *L. chinensis* seeds were reliable (Figure 4).

### 2.4. Metabolic Analysis of Seeds Treated with GA_3_

**Figure 5:**
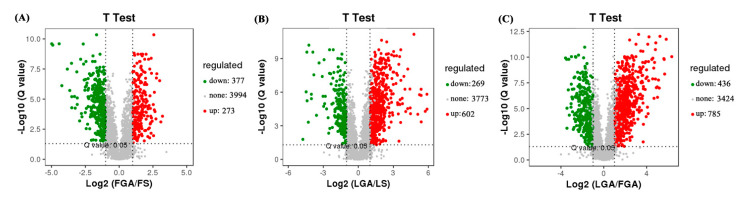
Volcano plot of differentially abundant metabolites in FGA vs. FS (**A**), LGA vs. LS (**B**) and LGA vs. FGA (**C**). The *x*-axis represents the log fold change, and the *y*-axis represents significance (*q*-value); green dots represent downregulated genes, and red dots represent upregulated genes.

To fully understand the metabolic changes that occur in response to GA_3_-mediated disruption of seed dormancy in *L. chinensis*, a nontarget metabolic analysis was performed using UPLC-qTOF-MS, and principal component analysis (PCA) of the whole samples (Figure S1A) showed that the same treatments were gathered together, indicating good repeatability between samples, while different treatments were separated from each other, indicating that there were different effects on metabolites between treatments. Each treatment group was separated by the first component (PC1), which means that the treatment was the most important factor causing differences in metabolites rather than random errors (Figure S1B,C). To understand the effects of the differentially abundant metabolites of GA_3_ on the germination of *L. chinensis* seeds, we identified 650 and 871 significantly different metabolites in FGA vs. FS and LGA vs. LS, respectively (Figure 5A,B). In addition, 1221 significantly different metabolites were also screened out in LGA vs. FGA to consider the influence of the hulls (Figure 5C).

**Figure 6:**
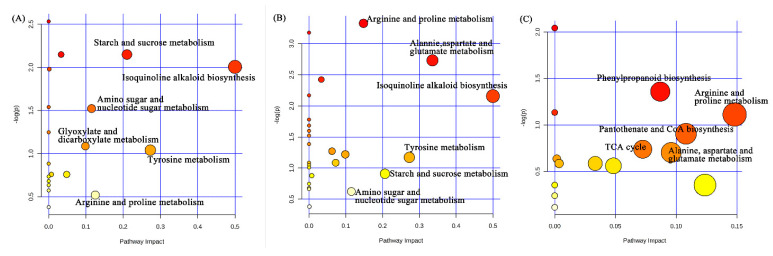
Pathway enrichment analysis of differentially abundant metabolites in FGA vs. FS (**A**), LGA vs. LS (**B**) and LGA vs. FGA (**C**). The size and colour of the bubbles represent the pathway impact and *p* value (−log(p)) of the enrichment analysis, respectively; the darker the colour is, the more significant the enrichment.

**Figure 7:**
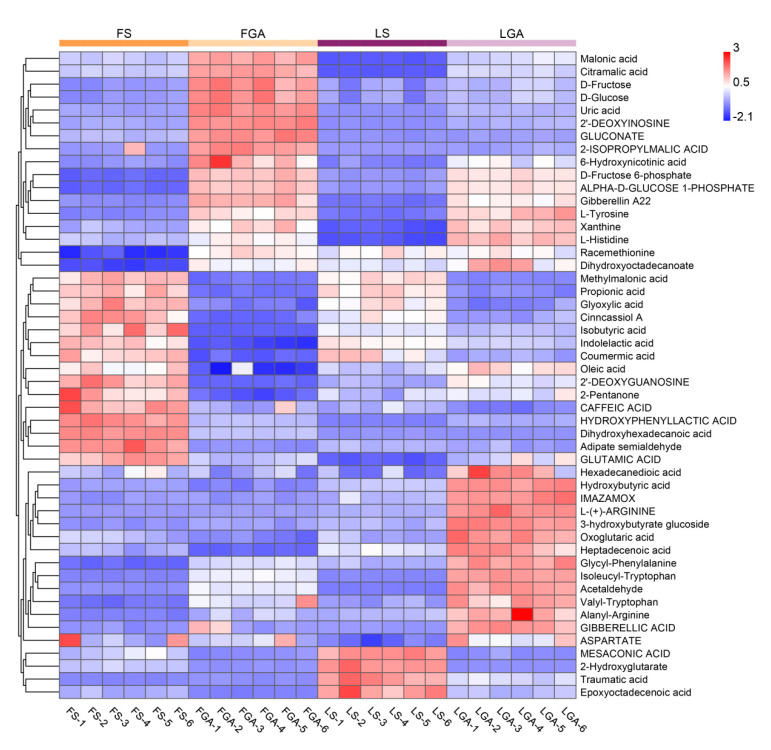
Clustering heatmap analysis of the main differentially abundant metabolites in the FS, FGA, LS and LGA treatment groups. Red indicates a higher abundance, and blue indicates a lower abundance.

Comparative analysis of the treatments of hulled seeds of *L. chinensis* with GA_3_ and distilled water showed a significant difference in metabolites, and the significantly enriched pathways included isoquinoline alkaloid biosynthesis, tyrosine metabolism, starch and sucrose metabolism, arginine and proline metabolism, amino sugar and nucleotide sugar metabolism, and glyoxylate and dicarboxylate metabolism (Figure 6A). However, the main pathways in the de-hulled seeds included isoquinoline alkaloid biosynthesis; alanine, aspartate and glutamate metabolism; tyrosine metabolism; starch and sucrose metabolism; arginine and proline metabolism; and amino sugar and nucleotide sugar metabolism (Figure 6B). Due to the differences in the main metabolic pathways associated with GA_3_ treatment of hulled and de-hulled seeds, the pathways associated with the hulls of *L. chinensis* seeds were also analysed (Figure 6C and Table S6). The main differentially abundant metabolite pathways were arginine and proline metabolism; pantothenate and CoA biosynthesis; phenylpropanoid biosynthesis; and alanine, aspartate and glutamate metabolism, which mainly synthesize some organic acids and amino acids, such as L-arginine, pantothenate and oxoglutaric acid. It can be seen from the clustering heat map analysis (Figure 7) of the main differentially abundant metabolites and the data in Table S7 that the abundance of the metabolites was significantly affected in hulled and de-hulled seeds of *L. chinensis* after soaking in GA_3_. Compared with those of seeds soaked in water, the contents of malonic acid and citramalic acid significantly increased in seeds treated with GA_3_ (2.16- and 2.18-fold in FGA; 16.68- and 34.17-fold in LGA). The levels of carbohydrates such as D-fructose, D-fructose 6-phosphate, D-glucose and D-glucose 1-phosphate were significantly increased in FGA, and they were also increased in LGA. In addition, the levels of most amino acids, such as L-tyrosine, L-histidine and L-arginine, were significantly increased in LGA, while the number of significantly enriched amino acids decreased in FGA.

### 2.5. Integrative Analysis of DEGs and Metabolites Involved Starch and Sucrose Metabolism in Seeds Treated with GA_3_

**Figure 8:**
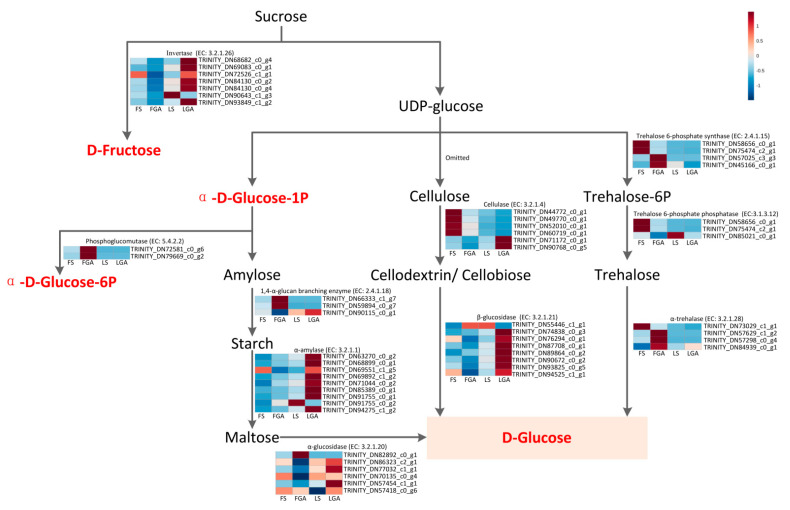
Starch and sucrose pathway analysis. The metabolic pathway was based on the KEGG database. Significantly differentially accumulated metabolites are indicated in bold and red, and the heatmap represents the gene expression levels in the four groups.

Starch and sucrose metabolism can provide energy and carbon skeletons for biosynthesis during seed germination. Moreover, sucrose can also act as a transport compound that is produced in the endosperm and moves into the embryo. GA_3_ treatment significantly influenced starch and sucrose metabolism in this study. Therefore, we performed an association analysis of DEGs and metabolites (α-D-glucose-1P, D-fructose, D-glucose and α-D-glucose-6P) in the starch and sucrose metabolic pathways (Figure 8). According to the results, 5 DEGs encoding α-glucosidase (*XYL1*) and 6 DEGs encoding invertase (*Inv*) were upregulated in de-hulled seeds after treatment with GA_3_, but most of these DEGs were downregulated in hulled seeds. At the same time, two DEGs, namely, *PGM2* (*TRINITY_DN79669_c0_g2*) and *PGM* (*TRINITY_DN72581_c0_g6*), encoding phosphoglucomutase, were upregulated in hulled seeds but exhibited very low expression in de-hulled seeds. In the process of starch hydrolysis, 2 and 1 DEGs encoding 1,4-α-glucan branching enzymes were significantly upregulated in FGA and LGA, respectively. In addition, 9 DEGs encoding α-amylase (*AMY1.1*, *AMY1.2* and *AMY1.6*) were significantly upregulated, and among them, *AMY1.1* (*TRINITY_DN91755_c0_g1*) had the highest expression level, which was 18.05 and 3.40 times higher in hulled and de-hulled seeds, respectively, under the GA_3_ treatment than under the control treatment. Most DEGs encoding cellulase were downregulated under GA_3_ treatment in hulled and de-hulled seeds compared with the seeds treated with sterile water, while 7 of 8 DEGs encoding β-glucosidase (*GLIP5*) showed significantly enhanced expression in de-hulled seeds. In addition, the trehalose 6-phosphatase synthase genes *tpsA* (*TRINITY_DN58656_c0_g1*) and *SPAC2E11.16c* (*TRINITY_DN75474_c2_g1*) and the trehalose 6-phosphatase phosphatase gene *TPP2* (*TRINITY_DN85021_c0_g1*) were all significantly downregulated in hulled and de-hulled seeds, while 3 DEGs encoding α-trehalase (treh) were upregulated.

**Figure 9:**
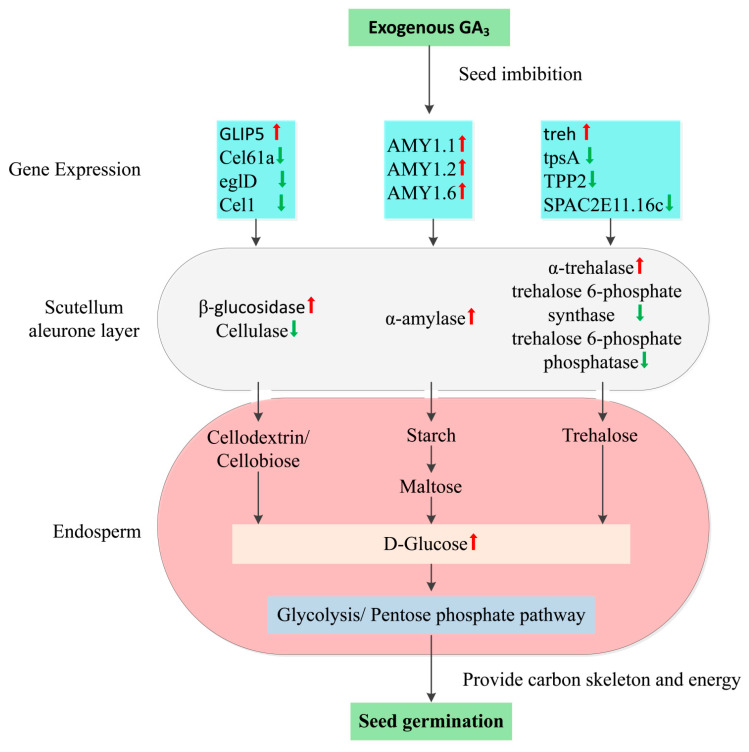
Schematic diagram summarizing the possible mechanism of GA_3_-mediated seed dormancy release. The red-up arrows indicate genes and metabolites that were upregulated, and the blue-down arrows indicate genes and metabolites that were downregulated.

We also constructed a diagram of the regulatory network to clearly depict the mechanism of GA_3_-mediated seed dormancy release through starch and sucrose metabolism using the DEGs and metabolites with similar regulatory trends in both FGA vs. FS and LGA vs. LS (Table S8). As shown in Figure 9, exogenous GA_3_ disrupted seed dormancy and promoted germination by promoting the expression of *AMY1.1*, *AMY1.2*, *AMY1.6, treh* and *GLIP5* and inhibiting the expression of factors related to cellulose (*Cel61a, eglD* and *cel1*), *tpsA*, *SPAC2E11.16c* and *TPP2*. Differential expression of these genes promoted the synthesis of α-amylase, β-glucosidase and α-trehalase in the scutellum and aleurone layers and reduced cellulose and trehalose 6-phosphate synthase/phosphatase synthesis. These hydrolases were then secreted into the endosperm to catalyse the hydrolysis of cellobiose, cellodextrin, starch, maltose and trehalose to produce glucose and ultimately provide carbon skeletons and energy for seed germination via glycolysis or the pentose phosphate pathway.

## 3. Discussion

The germination rate reflects the dynamic relationship between the seed germination rate and time. Germination potential is an indicator that reflects and explains the germination speed of seeds and can accurately reflect whether or not the seeds germinate in an orderly manner. The germination index is an indicator of whether the germination rate is consistent. Generally, the higher the germination potential and germination index are, the better the germination regularity and germination rate of the seeds. Light and temperature are two key factors affecting seed germination. To explore the effects of GA_3_ on seed germination of *L. chinensis* separately, we adopted consistent germination conditions and did not consider the interactive effects between light, temperature and GA_3_. Based on the whole germination process, GA_3_ treatment significantly promoted the germination rate of hulled and de-hulled *L. chinensis* seeds, and the best effect was obtained at a GA_3_ concentration of 577 μM (Figure 1). Therefore, GA_3_ treatment can significantly promote the germination of *L. chinensis* seeds, breaking dormancy; research by Cui [30] also verified these results. In addition, the hulls act as an obstacle to the process of seed germination in *L. chinensis*, and in the case of exogenous GA_3_ addition, the hulls also represent an obstacle. After removing the hulls, the germination rate of the *L. chinensis* seeds significantly improved, which is consistent with the research of Ma, et al. [31].

Seed germination is a complex biological process regulated by a large number of genes. In a suitable environment, seeds restore metabolic activity, and the enzymes and metabolites stored in the seeds are rapidly activated. After the absorption of water, this series of processes involves the regulation of a large number of genes and the energy supply [32]. In this study, we performed a transcriptomic analysis of hulled and de-hulled seeds treated with GA_3_ and distilled water and generated a total of 37,208 genes. Based on the GO annotation results, the oxidation-reduction process (GO: 0055114) and cellular component (GO: 0005575) terms were both significantly enriched in FGA vs. FS and LGA vs. LS (Table S4). It has been reported that energy metabolism mediated by redox activity may be conducive to effective metabolism during early seed germination [33]. In addition, cellular components (such as membrane, ribosome and nucleoplasm.) are also closely related to cell division, elongation or radicle emergence during seed germination. Moreover, the KEGG pathway analysis showed that protein processing in the endoplasmic reticulum, spliceosome, starch and sucrose metabolism, endocytosis and ribosome were significantly enriched pathways in both LGA vs. LS and FGA vs. FS (Figure 3), indicating that these pathways play an important role in promoting seed germination under GA_3_ treatment.

Seed hulls are one of the key factors that restrict seed germination, and the mechanical properties of seed hulls may represent an obstacle to the exchange of gas and water [34]. He et al. [35] mentioned in their study on seed dormancy in *L. chinensis* that seed hulls accounted for 28.4% of the causes of dormancy induction, and hulled seeds treated with GA_3_ also exhibited a significantly reduced percentage of seed dormancy. Therefore, in this study, the main metabolic pathways involved in the treatment of hulls after GA_3_ treatment and the main differentially abundant metabolites involved were explored. Comparison of the GA_3_-treated hulled and de-hulled seeds (Figure 6 and Table S6) showed that the main differentially abundant metabolites in hulls were L-arginine and feruloylputrescine in the pathway of arginine and proline metabolism, and 2-dehydroepianate and pantothenic acid in the pathway of pantothenate and CoA biosynthesis, which are key metabolites of synthetic organic acids. Similar findings were also reported by Yu [36], who studied the substances inhibiting seed germination and seedling growth in various parts of *Taxus chinensis* var. *mairei* seeds and found that organic acids, esters and alcohols are distributed in seed hulls. Similarly, germination-inhibiting organic acids were also detected in *Torreya grandis* seed hulls [37]. The organic acids in the hulls of seeds regulate the seed dormancy mechanism through the metabolism of arginine and proline and the synthesis of pantothenic acid and coenzyme A. In addition, arginine is an important N storage and transport amino acid and 17% of the total N content is arginine in seeds [38,39]. Arginine is first hydrolysed to ornithine and urea under the action of arginase during germination, and then urea is hydrolysed to NH_4_^+^ by urease [38,40]. The latter can be used to synthesize glutamine or glutamate and then participate in the tricarboxylic acid (TCA) cycle. In this study, the TCA cycle also occurred in the hulls treated with GA_3_, which may be because GA_3_ treatment provided energy for reducing the synthesis of germination inhibitors in the hulls.

Sucrose and starch are used to fuel the Krebs cycle to produce ATP/NADH and as substrates for biosynthesis during the process of dormancy release and germination, including for the synthesis of DNA and cell walls. The increase in the contents of some sugars during seed imbibition indicates the need for an increased energy supply [41]. Our results showed that starch and sucrose metabolism was a significantly enriched pathway in seeds treated with GA_3_ and the main metabolites that exhibited increased levels in the hulled and de-hulled seeds treated with GA_3_ were α-D-glucose-1P, D-fructose, D-glucose and α-D-glucose-6P, which may eventually participate in glycolysis, the TCA cycle or the pentose phosphate pathway. This suggested that exogenous GA_3_ could increase the energy supply for seeds by regulating starch and sucrose metabolism during the process of dormancy release. According to our results (Figure 8), GA_3_ promoted upregulation of the expression of the genes α-glucosidase (*XYL1*) and invertase (*Inv*) in de-hulled seeds of *L. chinensis*. Sucrose was hydrolysed to D-fructose via the action of *Inv*, and maltose could be hydrolysed to glucose by *XYL1*, but the expression of these two DEGs was downregulated in hulled seeds. At the same time, a downstream product of sucrose, α-D-glucose-1P, was converted to α-D-glucose-6P under the action of two upregulated DEGs, namely, *PGM2* (*TRINITY_DN79669_c0_g2*) and *PGM* (*TRINITY_DN72581_c0_g6*), in hulled seeds, but the expression of these DEGs was very low in de-hulled seeds; these DEGs encode phosphoglucomutase and are further involved in the glycolysis pathway. It has been reported that sucrose may be an intermediate of plant metabolism, and the decrease in sucrose decomposition results in low levels of glucose and fructose and inhibits seed germination [42].

In addition to sucrose, the decomposition of starch, cellulose and trehalose also provides energy and acts as the substrate for biosynthesis during seed germination. Starch is a polysaccharide stored in plant seeds that can be converted into reducing sugars (such as maltose and glucose) by amylase and plays an important role in seed germination [43]. The seed embryo needs nutrition during the development of seed morphology, and these nutrients are obtained not only by transformation of storage substances in the endosperm but also via the metabolism of nutrients in the embryo, which provides the energy needed for embryonic development. Li, et al. [44] reported that the starch content in the endosperm is dynamic, and starch is hydrolysed to sugar by amylase, providing energy for seed germination. Amylase not only converts starch to glucose and other reducing sugars by acting on the α-1,4-glycosidic bond but also reduces the viscosity of starch [45,46]. In this study, 9 DEGs encoding α-amylase (*AMY1.1*, *AMY1.2* and *AMY1.6*) were significantly upregulated, and among them, *AMY1.1* (*TRINITY_DN91755_c0_g1*) was upregulated 18.05- and 3.40-fold in hulled and de-hulled seeds, respectively. In the initial stages of germination, GA_3_ in embryos or exogenous first enters aleurone layer cells and promotes the expression of hydrolase genes by activating the signal transduction pathway. Amylase and other hydrolytic enzymes synthesized in the endoplasmic reticulum are processed by the Golgi apparatus and then secreted into the endosperm to function [47]. Under the action of α-amylase and the 1,4-α-glucan branching enzyme, amylose and starch stored in seeds can be hydrolysed to maltose and subsequently broken down to glucose by α-glucosidase. The α-amylase gene is a downstream target gene for GA-mediated regulation of seed germination and encodes an enzyme that hydrolyses starch in the endosperm. GA can induce the expression of the amylase gene and then promote starch hydrolysis via the GA response element [48]. Studies have shown that the transcription level of the α-amylase gene can be regulated, and GAMyb-type transcription factors play a key role in this process. GA regulates the expression of *GAMyb* in a DELLA-dependent manner, and *GAMyb* binds to the GA response element on the promoter of the α-amylase gene and activates its expression [49].

Cellulose is a key component of the plant cell wall. Cellulase cleaves cellulose to form cellobiose or cellodextrin, and then, β-glucosidase finally cleaves these two hydrolysates to glucose [50]. In this study, most DEGs encoding cellulase were downregulated under treatment with GA_3_ in hulled and de-hulled seeds compared with the group treated with sterile water, which inhibited the decomposition of cellulose; however, 7 of 8 DEGs encoding β-glucosidase (*GLIP5*) showed significantly enhanced expression in de-hulled seeds, breaking down cellodextrin and cellobiose to glucose. This is likely because elongation of the radicle cannot occur without cell wall remodelling, and on the other hand, decomposition of cellodextrin and cellobiose could also produce more D-glucose.

Trehalose is a nonreducing disaccharide that can be used as an energy source for glycolysis and is associated with various types of stress tolerance. The trehalose 6-phosphatase synthase gene and α-trehalase gene have opposite functions, regulating trehalose synthesis and degradation, respectively [51]. In seeds of *L. chinensis* after GA_3_ treatment, the trehalose 6-phosphatase synthase genes *tpsA* (*TRINITY_DN58656_c0_g1*) and *SPAC2E11.16c* (*TRINITY_DN75474_c2_g1*) and the trehalose 6-phosphatase phosphatase gene *TPP2* (*TRINITY_DN85021_c0_g1*) were all significantly downregulated, which inhibited the synthesis of trehalose. In addition, 3 DEGs encoding α-trehalase (*treh*) were upregulated, hydrolysing trehalose to glucose. A study on *Medicago truncatula* showed that the trehalose content decreased during seed imbibition and may be an important energy source compared to osmoprotectants [41]. A study on some fungal species also observed decreased trehalose content during spore germination [52]. Thus, trehalose may act as a buffer regulating the intracellular level of glucose and may begin to degrade when the intracellular glucose concentration is insufficient [53].

## 4. Materials and Methods

### 4.1. Plant Materials and Seed Treatments

Mature *L. chinensis* seeds of mixed populations were collected from the Songnen Grassland with the permission of the Grassland Station of Daqing in Heilongjiang Province in China in July 2017. The Heilongjiang Frigid Zone Plant Gene Resource Research Center undertook the formal identification of the samples and stored them at −5 °C with 50% relative humidity. The experiment was carried out in June 2018, and seeds were divided into two types: hulled seeds and de-hulled seeds (hulls were peeled off by hand). All seeds were disinfected for 5 min in 5% sodium hypochlorite, followed by rinsing with 75% alcohol twice. After rinsing three times using sterile water, the seeds were dried at room temperature.

The hulled and de-hulled seeds (100 mg per sample) were soaked in 50 mL of GA_3_ solution at different concentrations (289, 577 and 866 μM) for 24 h at 25 °C, and the control groups were soaked in sterile water. The concentration gradient of GA_3_ was based on a previous study [54]. All treatments were performed three times.

### 4.2. Seed Germination Assays

A total of 100 mg of sterilized seeds (approximately 50 seeds) were evenly placed in each petri dish. The petri dishes were placed in a room at 28 °C (light) or 19 °C (dark) for 12 h, and the filter paper was kept moist by watering regularly during germination. Seeds were considered to have germinated when the root tip protruded from the seed. The number of germinated seeds was counted daily for 21 days to calculate the germination rate for the first 7 days and the total germination rate. The germination potential was the germination number on the 6th day/the total number of seeds, and the germination index was calculated by the following equation (where Gt is the germination number at different times (7 days), and Dt is the number of days of germination) [55]:

Germination index = ∑Gt/Dt(1)

### 4.3. Samples for Transcriptomic and Metabolomic Analyses

The concentration of GA_3_ (577 μM) used was determined from the former germination test. Mature and plump seeds of *L. chinensis* with and without hulls were first disinfected and then soaked in 577 μM GA_3_ solution for 24 h, defined as FGA and LGA, respectively; another set of seeds was soaked in sterile water for 24 h, defined as FS and LS, respectively. After the treatments, seeds were placed in petri dishes to germinate at room temperature for 72 h. All the treatments were performed three times. Transcriptomic and metabolomic analyses were carried out by LC-Bio Technologies (Hangzhou) Co., Ltd (Hangzhou, China).

### 4.4. RNA Extraction, Quality Control and RNA-Seq

The OminiPlant RNA Kit (CWBIO, Beijing, China) was used to extract total RNA from each sample following the manufacturer’s procedure. Total RNA was checked for quantity and purity using a Bioanalyzer 2100 and an RNA 1000 Nano LabChip Kit (Agilent, Santa Clara, CA, USA) with RIN number > 7.0. Two rounds of purification were used to purify poly(A) RNA from 5 μg of total RNA using poly-T oligo-attached magnetic beads. Then, the mRNA was fragmented into small fragments using divalent cations at a high temperature. The cDNA library was created via reverse transcription using the mRNA-Seq Sample Preparation Kit (Illumina, San Diego, CA, USA), and paired-end libraries were constructed with an average insert fragment size of 300 bp (±50 bp). Finally, paired-end transcriptome sequencing of *L. chinensis* seeds was performed on an Illumina HiSeq4000 platform (San Diego, CA, USA) using the recommended protocols.

### 4.5. De novo Assembly and Functional Annotation

The reads in the sequencing data that contained low-quality bases, adaptor contamination, and undetermined bases were first removed using cutadapt [56] and in-house Perl scripts. Then, FastQC (http://www.bioinformatics.babraham.ac.uk/projects/fastqc/, accessed on 19 September 2018) was used to check the sequence quality, including the Q20, Q30 and GC content of the clean data. All further analyses in this study were based on high-quality clean data. Trinity (version 2.4.0) [57] was employed for de novo assembly of our transcriptomic data and for grouping transcripts into clusters on the basis of shared sequence content. Each transcript cluster was very loosely defined as a ‘gene’, and the longest transcript in the cluster was taken as the ‘gene’ sequence.

DIAMOND [58] was used with an E-value threshold < 0.00001 to annotate these assembled unigenes by alignment against the nonredundant protein (Nr) (http://www.ncbi.nlm.nih.gov/, accessed on 19 September 2018), GO (http://www.geneontology.org, accessed on 19 September 2018), SwissProt (http://www.expasy.ch/sprot/, accessed on 19 September 2018), KEGG (http://www.genome.jp/kegg/, accessed on 19 September 2018) and eggnog (http://eggnogdb.embl.de/, accessed on 19 September 2018) databases. Transcript per million (TPM) [59] values were calculated using Salmon [60] to reflect the unigene expression levels. Differentially expressed unigenes were screened using the R package edgeR [61] with the criteria of FDR ≤ 0.05, log2 (fold change) > 1 or log2 (fold change) < −1. Then, GO and KEGG pathway enrichment analyses of differentially expressed unigenes were carried out again by using in-house Perl scripts.

### 4.6. Validation of Transcriptomic Data for Real-Time Quantitative Reverse Transcription PCR (qRT-PCR)

To verify the accuracy of our transcriptomic data, ten DEGs involved in *L. chinensis* seed dormancy release were selected for qRT-PCR, and the specific primers for these genes were designed with Primer 6.0 (Table S5). The synthesis of first-strand cDNA was performed using the TUREscript 1st Strand cDNA Synthesis Kit (Aidlab, Beijing, China). The reaction contained cDNA (1000 ng), 5× RT Reaction Mix (4 μL), random primer/oligodT (0.8 μL), and TUREscript H^-^ RTase/RI Mix (0.8 μL), and RNase Free dH_2_O was added to obtain a final volume of 20 μL. The qRT-PCR contained 5 μL of 2× SYBR^®^ Green Supermix (Vazyme, Nanjing, China), 0.5 μL of forward primer, 0.5 μL of reverse primer, 1 μL of cDNA, and 3 μL of ddH_2_O. qRT-PCR was conducted on an Analytik Jena qTOWER2.2 instrument (Jena, Germany) with the following program: 95 °C for 3 min, followed by 39 cycles of 95 °C for 10 s and 58 °C for 30 s. All the samples were examined in triplicate with four technical replicates. The relative expression of genes was calculated by the 2^−ΔΔCt^ method using actin as the reference gene [62,63].

### 4.7. Metabolite Extraction and Metabolic Spectrum Analysis

The collected samples were first thawed on ice, and then, 120 μL of precooled 50% methanol buffer was added to 20 μL of sample followed by 1 min of vortexing and 10 min of incubation at room temperature. The extraction mixture solution was stored overnight at −20 °C in a freezer and then centrifuged at 4000 r/min for 20 min. The supernatants were transferred into 96-well plates and stored at −80 °C for subsequent LC-MS analysis. Furthermore, 10 μL of each extraction mixture was combined into a pooled quality control (QC) sample.

In this study, 43 samples of *L. chinensis* seeds (including QC samples) were detected with a TripleTOF 5600 system (ABSCIEX, Foster City, California, USA) in positive and negative ion modes, and the mass spectrum data were interpreted in combination with biological information analysis. The biological information analysis mainly used XCMS software (https://github.com/sneumann/xcms, accessed on 21 August 2018) for peak extraction and QC of peak extraction. MetaX software [64] was used to screen quantitative and differentially abundant substances. Metabolites were annotated in HMDB, KEGG and other databases.

The first-level mass spectrometry information was used for identification, and the second-level mass spectrometry information was used for matching with the in-house standard database. In this paper, the original mass spectrometry data were transformed to the readable mzXML data format by using the msconvert tool of Proteowizard software [65].

### 4.8. Metabolic Pathway Construction

Three-dimensional data obtained in this study, including sample name, peak number and normalized data, were input into SIMCA software (V14, Umetrics AB, Umea, Sweden), and PCA was performed for all samples. Student’s *t*-test (*p* < 0.05) and variable importance in the projection (VIP) values > 1 were used to search for differentially abundant metabolites. Metabolite pathways were constructed by searching noncommercial databases, such as KEGG (http://www.genome.jp/kegg/, accessed on 21 August 2018) and NIST (http://www.nist.gov/index.html, accessed on 21 August 2018).

### 4.9. Statistical Analysis

One-way ANOVA was conducted to compare the differences between treatments with different concentrations of GA_3_ using Duncan’s multiple comparisons test (*p* < 0.05) in SAS 9.0 (SAS Institute, Cary, NC, USA). An independent *t*-test was also conducted to examine the differences between de-hulled and hulled groups. Data are shown as the means ± standard deviation (*n* = 3).

## 5. Conclusions

In this study, through transcriptomics and metabolomics analysis, we analysed the molecular mechanism by which GA_3_ disrupts seed dormancy in *L. chinensis*. The results revealed that exogenous GA_3_ can significantly promote seed germination by regulating some important genes and their related metabolites. Starch and sucrose metabolism is one of the most highly enriched pathways and may play a key role in energy supply for the transition of *L. chinensis* seeds from the dormant state to germination by suppressing the expression of *Cel61a*, *egID*, *cel1*, *tpsA*, *SPAC2E11.16c* and *TPP2*, enhancing the expression of *AMY1.1*, *AMY1.2*, *AMY1.6* and *GLIP5*, and finally inhibiting the synthesis of cellobiose, cellodextrin, and trehalose, while promoting the hydrolysis of sucrose, starch, cellobiose, cellodextrin, and trehalose to glucose. These findings provide insights for understanding the mechanisms by which GA_3_ disrupts dormancy and provide valuable information for further breeding of *L. chinensis* varieties with high germination rates.

## Data Availability

The RNA-seq datasets generated during the current study have been submitted to the NCBI Sequence Read Archive under the accession number PRJNA642340 (https://www.ncbi.nlm.nih.gov/sra/PRJNA642340, accessed on 28 June 2020), and other data supporting the results are included in this published article and its Supplementary Information Files.

## Supplementary Materials

The following are available online at https://www.mdpi.com/article/10.3390/ijms22084161/s1.
