# Supplementary material for: Integrated Analysis of the Transcriptome and Metabolome Revealed Candidate Genes Involved in GA3-Induced Dormancy Release in Leymus chinensis Seeds
Source: Int J Mol Sci. 2021 Apr 17;22(8):4161. doi: 10.3390/ijms22084161 (PMC8074249; doi:10.3390/ijms22084161)
Supplement: Supplementary file 1 [file ijms-22-04161-s001.zip › Table S5.pdf]

Table S5 Primer sequences of candidate DEGs and reference genes used in RT-qPCR

Genes		Primers
actin	F	ATTGTGCTCAGTGGTGGGTCA
	R	CCAATCCAAACACTGTACTTCCTC
AMY1.2	F	TCCAAGTACGGCAACAAGG
	R	CGGTGGTTGATGACGATGT
AMY1.6	F	CGATGACAAGGTTATCACGAAGA
	R	TTCTCCCAGACGCAGTAATTG
BXL7	F	AGCCAGCAGCAGGAGAGC
	R	GGAGACGGCGGTGATGAGG
BACOVA_02659	F	CGACAACCTGAACCTGACGAT
	R	CCAGAGATGAGCACCACCAC
PME68	F	CCTCCGTAACGTTCTCT
	R	TTGTCCTTGTGACTCCT
CSLF6	F	GGCAAAGAAATCGGCTGGGTGTA
	R	ATGAAGGCGTGTGGGTAGATGGA
1-SST	F	AGGAGGCAGGAGTGTACTTGTTCA
	R	GGAGAGTTGGTTGTGTGCTGAGTC
BGLU30	F	GAGGTTGAGGATTGTTAT
	R	TTGATTGAAGTCTTAGAAAC
At5g42100	F	ACCTCCTCCCGTACATCT
	R	CGAAGTAGGGTAGGCATT
tpsA	F	GTTCCAAGGCAAGGTCGTCAC
	R	AGGGCTGTATCCGCTCTT
