# Supplementary material for: Integrated Analysis of the Transcriptome and Metabolome Revealed Candidate Genes Involved in GA3-Induced Dormancy Release in Leymus chinensis Seeds
Source: Int J Mol Sci. 2021 Apr 17;22(8):4161. doi: 10.3390/ijms22084161 (PMC8074249; doi:10.3390/ijms22084161)
Supplement: Supplementary file 1 [file ijms-22-04161-s001.zip › Table S6.pdf]

Table S6 Differentially abundant metabolites in major enriched pathways in LGA vs FGA

| Metabolite Pathway | Metabolite | $Log_2FC$ |
|---|---|---|
| Alanine, aspartate and glutamate metabolism | Oxoglutaric acid | 1.72 |
| | Succinate | 1.38 |
| Arginine and proline metabolism | N2-Succinyl-L-ornithine | 1.35 |
| | N-Succinyl-L-glutamate 5-semialdehyde | 1.74 |
| | N2-Succinyl-L-arginine | 2.01 |
| | L-Arginine | 3. 45 |
| | Feruloylputrescine | 2.08 |
| | 4-(L-gamma-Glutamylamino)butanoate | 1.35 |
| | S-Adenosylmethioninamine | -1.51 |
| Pantothenate and CoA biosynthesis | 2-Dehydropantoate | 1.09 |
| | (R)-4-Dehydropantoate | 1.09 |
| | Pantothenate | -2.38 |
| Phenylpropanoid biosynthesis | N1, N5, N10-Tricoumaroyl spermidine | 2.13 |
| | 5-O-Caffeoylshikimic acid | -1.66 |
| | Sinapate | -2.21 |
| | p-Coumaroyl quinic acid | 1.77 |
| | Coniferyl aldehyde | -2.30 |
| | Sinapoyl aldehyde | -2.34 |
| | 4-Coumaroylshikimate | -2.29 |
