# Supplementary figures and images for: Integrated Analysis of the Transcriptome and Metabolome Revealed Candidate Genes Involved in GA3-Induced Dormancy Release in Leymus chinensis Seeds
Source: Int J Mol Sci. 2021 Apr 17;22(8):4161. doi: 10.3390/ijms22084161 (PMC8074249; doi:10.3390/ijms22084161)

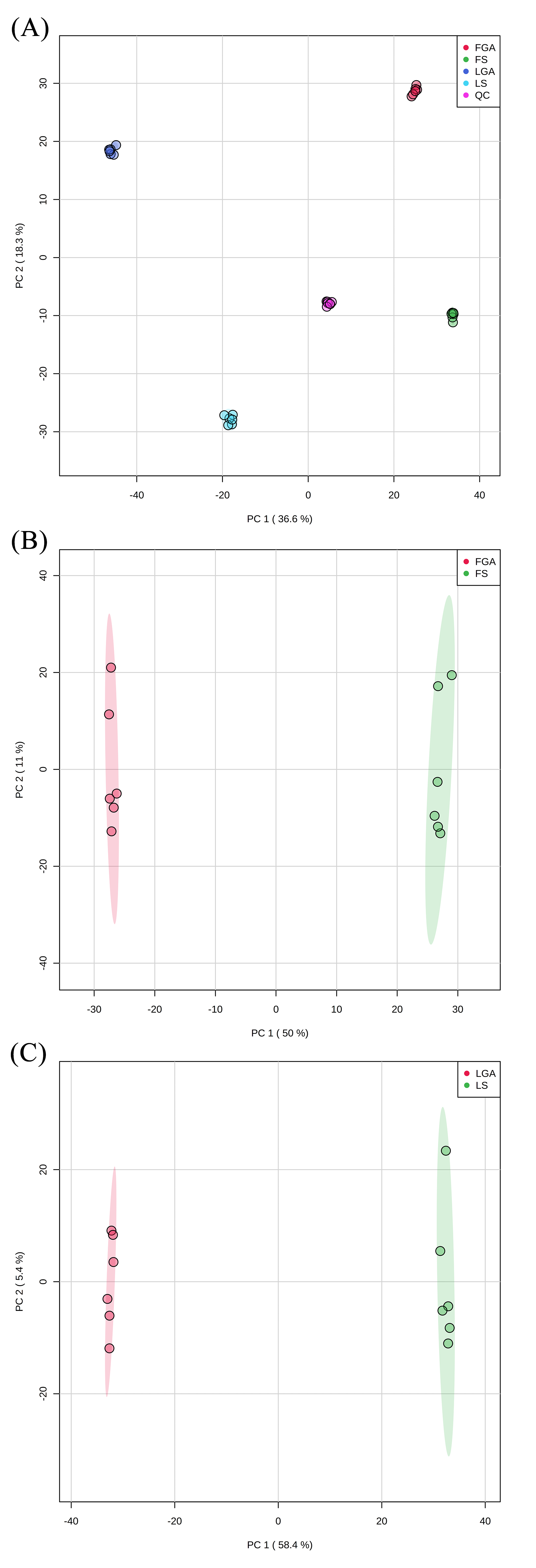

Supplement: Supplementary file 1 [file ijms-22-04161-s001.zip › Figure S1.tif]
